# Differences in diagnosis, treatment, and survival rate of acute myeloid leukemia with or without disabilities: A national cohort study in the Republic of Korea

**DOI:** 10.1002/cam4.3179

**Published:** 2020-06-03

**Authors:** Jihyun Kwon, So Young Kim, Kyoung Eun Yeob, Hye Sook Han, Ki Hyeong Lee, Dong Wook Shin, Yeon‐Yong Kim, Jong Heon Park, Jong Hyock Park, Ichiro Kawachi

**Affiliations:** ^1^ Division of Hematology‐Oncology Department of Internal Medicine Chungbuk National University Hospital Cheongju Korea; ^2^ Department of Internal Medicine College of Medicine Chungbuk National University Cheongju Korea; ^3^ Department of Public Health and Preventive Medicine Chungbuk National University Hospital Cheongju Korea; ^4^ College of Medicine/Graduate School of Health Science Business Convergence Chungbuk National University Cheongju Korea; ^5^ T. H. Chan School of Public Health Harvard University Boston MA USA; ^6^ Supportive Care Center/Department of Family Medicine Samsung Medical Center Sungkyunkwan University School of Medicine Seoul Korea; ^7^ Department of Digital Health SAIHST Sungkyunkwan University Seoul Korea; ^8^ Big Data Steering Department National Health Insurance Service Wonju Korea

**Keywords:** acute myeloid leukemia, cohort, disability, survival, treatment

## Abstract

We analyzed the patterns of diagnosis, treatment, and prognoses of acute myeloid leukemia (AML) patients with and without disabilities. The data were collected from the National Disability Database, the Korean Central Cancer Registry, and the Korean National Health Insurance claim database. We built a cohort of 2 776 450 people with disabilities and a nondisabled cohort of 8 329 350 people who were selected at a ratio of 1:3 by matching age and sex. From this population, adult patients who were diagnosed with AML were analyzed. The number of patients with AML were 26.74 per 100 000 in people without disabilities and 20.39 per 100 000 in those with disabilities (*P* < .0001). The proportion of AML patients receiving chemotherapy and those of patients receiving transplants were significantly lower in the disabled population than that of nondisabled populations (71.2% vs 77.1%, *P* = .0031, and 17.5% vs 26.9%, *P* = .002). This trend was more pronounced in subgroups of communication disability and major internal organ disorder. The median survival was 10.8 months for patients with disabilities, which was significantly shorter than 17.1 months for those without a disability (*P* = .002). Individuals with disabilities have a low diagnosis rate of AML and undergo less active treatment, which results in inferior prognosis.

## Graphical abstract

We report herein that individuals with disabilities have a relatively low diagnosis rate of acute myeloid leukemia (AML), undergo less active treatment, and have significantly lower survival rates than those without a disability.

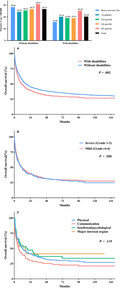

## 1 INTRODUCTION

Acute myeloid leukemia (AML) is the second most common hematologic malignancy in Korea.^1^ AML has several clinical features that distinguished it from solid cancer, including acute onset and rapid progression. Therefore, early detection and immediate treatment are essential to lowering mortality rate. In contrast, the cure rate of AML is relatively high compared to that of other malignant diseases, mainly due to high sensitivity to cytotoxic chemotherapy. Proper AML treatment should be accompanied by a high level of medical care, including from experienced hematologists, medical facilities capable of performing high‐dose chemotherapy and hematopoietic stem cell transplantation (HSCT), and general supportive care for infectious complications.

Survival outcomes of patients with AML have improved compared to past decades.^2^ Several factors, such as accumulation of experienced medical staff, development of potent antibiotics and antifungal drugs, and increased awareness of HSCT contributed to this progress. However, this improvement is limited to young and relatively fit patients; the prognosis of vulnerable patients such as older patients is not improving. Elderly patients with AML have poorer prognosis than young patients due to unfavorable cytogenetic or molecular features, several comorbidities, and impossibility of intensive treatment.^3^, ^4^

Patients with disabilities are an important subgroup of vulnerable patients with malignancies; vulnerabilities of individuals with disabilities are more diverse than those of elderly individuals. In addition to major organ dysfunction, other dysfunctions include perceptual degradation, communication problems, mobility limitation, and mental function problems. In clinical trials, inclusion criteria for study treatment generally include only the functions of major internal organs, such as cardiac, renal, and hepatic functions. However, even if physically healthy, disabilities of these patients indirectly affect cancer diagnosis and treatment. Some researchers reported that cancer screening is less commonly performed in individuals with than those without disabilities; treatment for cancer is also less active in disabled individuals.^5^, ^6^, ^7^

Socioeconomic problems also affect individuals with disabilities. Disabilities are commonly associated with unemployed status, low income, and poverty.^8^, ^9^ Recent advances in the treatment of AML patients, including elderly patients, are largely due to the development of novel drugs and the progression of HSCT, although these have inevitably led to higher treatment costs. Therefore, it is unknown whether benefits concerning the recent development of AML treatment are equally provided to individuals of low socioeconomic status, including those with disabilities. Such a consideration will contribute to establishing policies regarding the distribution of health‐care costs and the provision of public health services.

Considering these facts overall, we assumed that people with disabilities are likely to have inferior outcomes compare to those without disabilities due to the inappropriate treatment AML in various ways, either due to the disability itself or due to the socioeconomic disadvantages associated with the disability. As few studies have examined how these dysfunctions affect AML diagnosis and treatment, we constructed a cohort of involving nearly two million disabled individuals using the disability registration system of the Republic of Korea and analyzed diagnosis rates, treatment patterns, and outcomes of AML. We also compared these data with those of patients without disabilities. Finally, we assessed the effects of the type and severity of disability on the AML clinical course.

## 2 METHODS

### 2.1 Data sources and case selection

We used three national databases; the Korean National Disability Database, Korean Central Cancer Registry (KCCR), and Korean National Health Insurance (KNHI) claims database. The National Disability Registry includes more than 90% of Korean individuals with disabilities and provides a variety of information including type and severity of disability.^10^ The KCCR is a nationwide government‐sponsored cancer registry, which includes demographic characteristics of cancer patients and information about their cancer status, such as site, pathology, and stage. The KNHI database contains information regarding the patients’ health insurance premiums, residential areas, definition and statuses of their disease, provided treatments, and outcomes.

We compiled cases involving 2 776 450 people with disabilities using disability registration data. In addition, frequency‐matched random sampling was performed with the people without disabilities using the merged database mentioned above, grouped by age and sex at a ratio of 1:3 (2 776 450 people with disabilities, 8 329 350 people without disabilities, for a total of 11 105 800 subjects). We found individuals diagnosed with AML in both cohorts. Exclusion criteria included patients aged <19 years, previously diagnosed with other cancers, with no information regarding health insurance premiums, and those diagnosed with AML before 2002 (when the KNHI began collecting claims data).

### 2.2 Variables

We collected demographic data including age, sex, disease, treatment strategy, and outcomes from the KCCR database. Details of the disability in each case were sourced from the Korean National Disability Database.^11^ KNHI provided information about each patient's economic situation, insurance coverage, the treatment provided, medical costs, and medical institution usage patterns.

We classified disability types into four categories: physical, communication, intellectual, or psychological, and those affecting major internal organs (Table S1). Disability severity was graded from 1 (very severe) to 6 (very mild) based on the degree of functional impairment as determined by a medical specialist. We then simplified the severity to two categories, severe (grade 1‐3) or mild (grade 4‐6). The economic status was estimated using the KNHI premium because it is calculated on the basis of income, property, and automobile taxes for each household. Economic status of patients was categorized as follows: below the poverty line (lowest income) and quintile I, II, III, and IV (highest income). The use of certain antileukemic drugs listed below from the KNHI database was also investigated. These were cytarabine, idarubicin, daunorubicin, mitoxantrone, etoposide, cyclophosphamide, vincristine, fludarabine, methotrexate, and decitabine. If there was a record of one or more anticancer drugs prescribed, the patient was defined as having received systemic chemotherapy.

### 2.3 Statistical analyses

A chi‐squared test was used to determine discrepancies in diagnosis rate and treatment behavior according to various factors including age, sex, economic status, and type and severity of disability. The analysis of the patterns of transplantation therapy was limited to patients aged <65 years because KNHI ensures coverage for HSCT only in young patients. Survival outcomes and related risk factors were evaluated using the Kaplan‐Meier method with the log‐rank test and a multivariate Cox proportional hazards regression analysis. Before the analysis, the proportionality assumption was confirmed by plotting log hazard estimates against observation periods. Each covariate used for adjustment had Schoenfeld residual values indicating the proportional‐hazard assumption was fulfilled (*P* > .1 for all covariates). All statistical analyses were performed using SAS software (version 9.4; SAS Institute, Inc); differences were considered statistically significant when two‐sided *P* ≤.05 were found. The study protocol was approved by the Institutional Review Board of Chungbuk National University (CBNU‐201607‐BM‐288‐01).

## 3 RESULTS

### 3.1 Baseline characteristics

Baseline characteristics of the population cohort are described in Table S2. Briefly, 33.7% of individuals were ≥65 years old and 58.5% were male. The economic situation of the cohort with disability was significantly worse. Among individuals with disabilities, approximately 60% of people had a mild (grade 4‐6) disability. Physical disabilities were the most common type (1 728 916, 62.3%) followed by communication disabilities (588 712, 21.2%), intellectual or psychological disabilities (307 720, 11.1%), and major internal organ disabilities (151 102, 5.4%). Individuals with a severe disability or intellectual/psychological disability were younger and of a lower income status (Table S3).

### 3.2 Discrepancies in AML diagnosis rate

Among 2793 eligible patients with diagnosed AML, 566 (20.3%) had at least one disability. Patients with AML and disabilities had higher mean Charlson Comorbidity Index (CCI) scores compared to those in the control group (2.43 vs 2.15, *P* < .001). The percentage of AML patients in the lowest income group was higher in the people with disabilities than those without disabilities (13.1% vs 4.1%, Table 1). Patients with AML and disabilities of severe degree had relatively poorer overall health conditions (as represented by the high CCI score) and lower income status compare to patients with mild disabilities. Patients with intellectual/psychological disabilities were younger and had a lower CCI score than patients with a disability of other type; moreover, the percentage of patients with the lowest income status was highest in this group (Table 1).

**Table 1:** Baseline characteristics of patients with AML with or without disabilities

	Patients without disabilities N = 2227	Patients with disabilities (N = 566)									
		Total	*P*	Grade of disability			Type of disability				
				Grade 1‐3 (severe) N = 179	Grade 4‐6 (mild) N = 387	*P*	Physical N = 393	Communication N = 118	Intellectual/ psychological N = 38	Major internal organs N = 17	*P*
Age											
Mean ± SD	64.20 ± 14.09	63.96 ± 14.13		59.56 ± 15.10	65.99 ± 13.19		64.20 ± 12.91	69.39 ± 12.68	45.15 ± 15.60	62.76 ± 12.94	
Age, group (n,%)											
19‐40	153 (6.9)	46 (8.1)	.3805	24 (13.4)	22 (5.7)	.0002	26 (6.6)	4 (3.4)	15 (39.5)	1 (5.9)	<.0001
41‐64	844 (37.9)	200 (35.3)		74 (41.3)	126 (32.6)		145 (36.9)	28 (23.7)	20 (52.6)	7 (41.2)	
65‐	1230 (55.2)	320 (56.5)		81 (45.3)	239 (61.8)		222 (56.5)	86 (72.9)	3 (7.9)	9 (52.9)	
Sex (n,%)											
Male	1423 (63.9)	344 (60.8)	.1691	106 (59.2)	238 (61.5)	.6053	233 (59.3)	87 (73.7)	18 (47.4)	6 (35.3)	.001
Female	804 (36.1)	222 (39.2)		73 (40.8)	149 (38.5)		160 (40.7)	31 (26.3)	20 (52.6)	11 (64.7)	
CCI (n,%)											
Mean ± SD	2.15 ± 1.15	2.43 ± 1.21		2.65 ± 1.26	2.34 ± 1.19		2.48 ± 1.21	2.23 ± 1.19	2.00 ± 1.29	3.06 ± 1.12	
0	804 (36.1)	154 (27.2)	<.0001	59 (33.0)	95 (24.5)	.0155	108 (27.5)	27 (22.9)	18 (47.4)	1 (5.9)	.0005
1	557 (25.0)	128 (22.6)		34 (19.0)	94 (24.3)		81 (20.6)	35 (29.7)	11 (28.9)	1 (5.9)	
2	375 (16.8)	100 (17.7)		21 (11.7)	79 (20.4)		70 (17.8)	21 (17.8)	3 (7.9)	6 (35.3)	
3	204 (9.2)	60 (10.6)		18 (10.1)	42 (10.9)		45 (11.5)	14 (11.9)	1 (2.6)	0 0.0	
4	287 (12.9)	124 (21.9)		47 (26.3)	77 (19.9)		89 (22.6)	21 (17.8)	5 (13.2)	9 (52.9)	
Income status (n,%)											
Below poverty line	92 (4.1)	74 (13.1)	<.0001	39 (21.8)	35 (9.0)	<.0001	43 (10.9)	14 (11.9)	14 (36.8)	3 (17.6)	.0019
1st quartile	359 (16.1)	104 (18.4)		39 (21.8)	65 (16.8)		78 (19.8)	16 (13.6)	7 (18.4)	3 (17.6)	
2nd quartile	374 (16.8)	89 (15.7)		30 (16.8)	59 (15.2)		55 (14.0)	27 (22.9)	6 (15.8)	1 (5.9)	
3rd quartile	517 (23.2)	110 (19.4)		28 (15.6)	82 (21.2)		81 (20.6)	20 (16.9)	5 (13.2)	4 (23.5)	
4th quartile	885 (39.7)	189 (33.4)		43 (24.0)	146 (37.7)		136 (34.6)	41 (34.7)	6 (15.8)	6 (35.3)	

Abbreviations: AML, acute myeloid leukemia; CCI, Charlson Comorbidity Index; N, number; SD, standard deviation.

The overall AML diagnosis rate was 25.15 per 100 000. The number of patients with diagnosed with AML was significantly lower in those with compare to those without disabilities (20.39 vs 26.74 per 100 000, *P* < .0001, Table 2). This trend was observed regardless of the age group tested. Under the age of 65, there were 18.7 per 100 000 patients without disabilities and 13.3 per 100 000 patients with disabilities. At age 65 and older, there were 45.2 per 100 000 patients without disabilities and 34.8 per 100 000 patients with disabilities. The diagnosis rate of AML was much lower in patients with severe disabilities compared to those with mild disabilities. (16.4 vs 22.97 per 100 000, *P* < .0001). In addition, the diagnosis rate was lowest in those with disabilities of the major internal organs, followed by those with intellectual/psychological disabilities.

**Table 2:** Comparative analysis of diagnosis rate of AML by disability, degree of disability, and type of disability

Patient group	Diagnosis rate (patient N per 100 000)	*P*
Patients without disabilities	26.74	<.0001
Patients with a disability	20.39	
Severity of disability		
Severe (grade 1‐3)	16.4	<.0001
Mild (grade 4‐6)	22.97	
Type of disability		
Physical	22.73	<.0001
Communication	20.24	
Intellectual or psychological	12.35	
Major internal organs	11.25	

Abbreviations: AML, acute myeloid leukemia; N, number.

**Figure 1:**
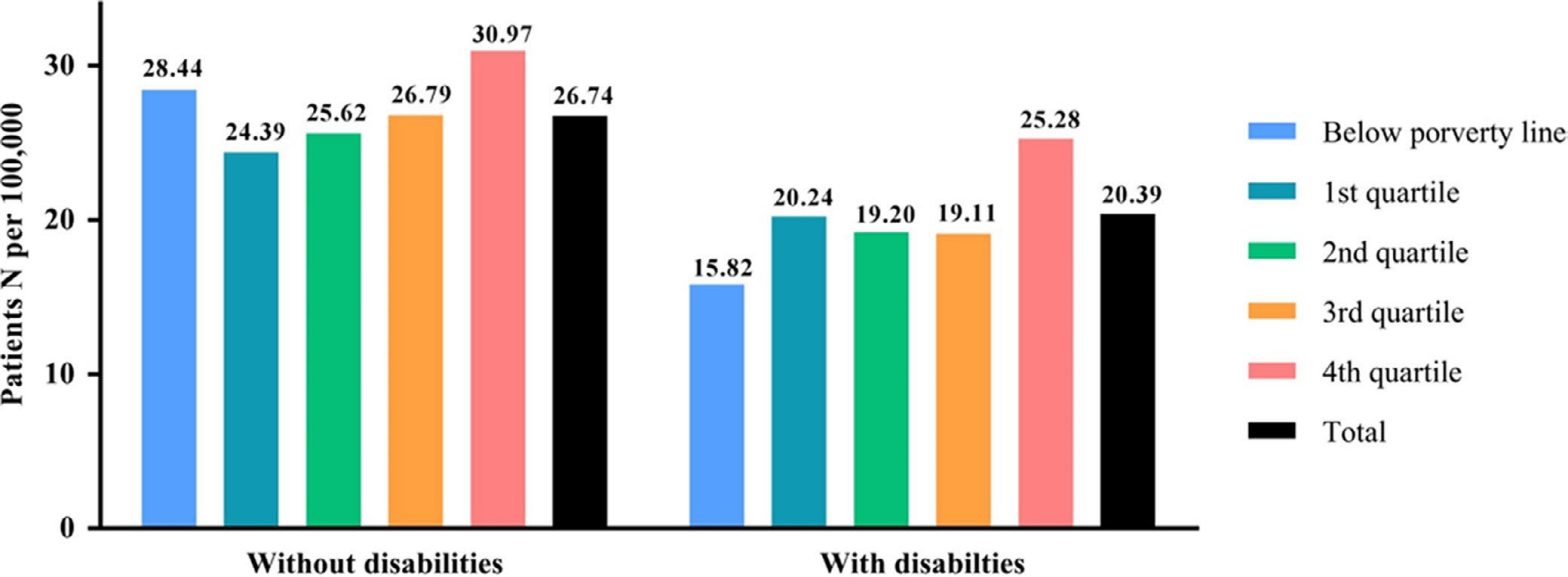
The number of AML patients per million, comparison by income level in each cohort. AML, acute myeloid leukemia; N, number

The impact of a low‐income status on the AML diagnosis rate was significant among individuals with disabilities. The diagnosis rate of AML in the lowest income group was 28.44 per 100 000 individuals without a disability, which was not lower than the overall diagnosis rate. However, the diagnosis rate of individuals with disabilities in the lowest income group was lowest (15.82 per 100 000), while the highest income group had the highest diagnosis rate (25.28 per 100 000) (Figure 1).

### 3.3 Analysis of treatment patterns for AML

Systemic chemotherapy for AML was administered to 75.9% of all patients. Patients with AML and disability had significantly lower rates of chemotherapy administration than those without disabilities (71.2% vs 77.1%, *P* = .0031, Table 3). The median number of anticancer drugs used for patients with disabilities was 2.75, which was significantly lower than the rate of 2.98 for those without a disability (*P* = .0075). The severity of the disability did not affect the chemotherapy rate. However, the rates of chemotherapy by disability type were significantly different. Patients with communication (61.9%) and major internal organ disabilities (64.7%) were less frequently treated for AML compare to those with physical (73.3%) or intellectual/psychological disabilities (81.6%, *P* = .0422). The median value of anticancer drugs used for patients with communication disorders (2.61) was nonsignificantly lower than those of other disorders (2.77 for physical, 2.90 for intellectual/psychological, and 2.72 for major internal organ disabilities).

**Table 3:** Comparative analysis of treatment behavior by disability, degree of disability, and type of disability

Patient group	Systemic chemotherapy		Allogeneic hematopoietic stem cell transplantation	
	%	*P*	%	*P*
Patients without disabilities	77.1	.0031	26.9	.0027
Patients with a disability	71.2		17.5	
Severity of disability				
Severe (grade 1‐3) disability	70.9	.0075	13.3	.1570
Mild (grade 4‐6) disability	71.3		20.3	
Type of disability				
Physical	73.3	.0422	18.1	.0204
Communication	61.9		15.6	
Intellectual or psychological	81.6		17.1	
Major internal organs	64.7		12.5	

Among the 1243 patients who were younger than 65 years, allogeneic HSCT (allo‐HSCT) was performed in 311 patients (25.0%). Among those without disabilities, 26.9% (268 patients) received allo‐HSCT, which was significantly higher than the 17.5% (43 patients) in those with disabilities (*P* = .0027, Table 3). Among patients with disabilities, nonsignificantly fewer patients with disabilities of a severe degree received allo‐HSCT compared to patients with mild disabilities (13.3% vs 20.3%, *P* = .1570). The rate of receiving allo‐HSCT was highest in those with a physical disability (18.1%), followed by those with a intellectual/psychological disability (17.2%) and a communication disability (15.6%). The rate of transplantation in patients with major internal organ disorders (12.5%) was lower compared to the proportion in the entire cohort.

### 3.4 Survival analysis

**Figure 2:**
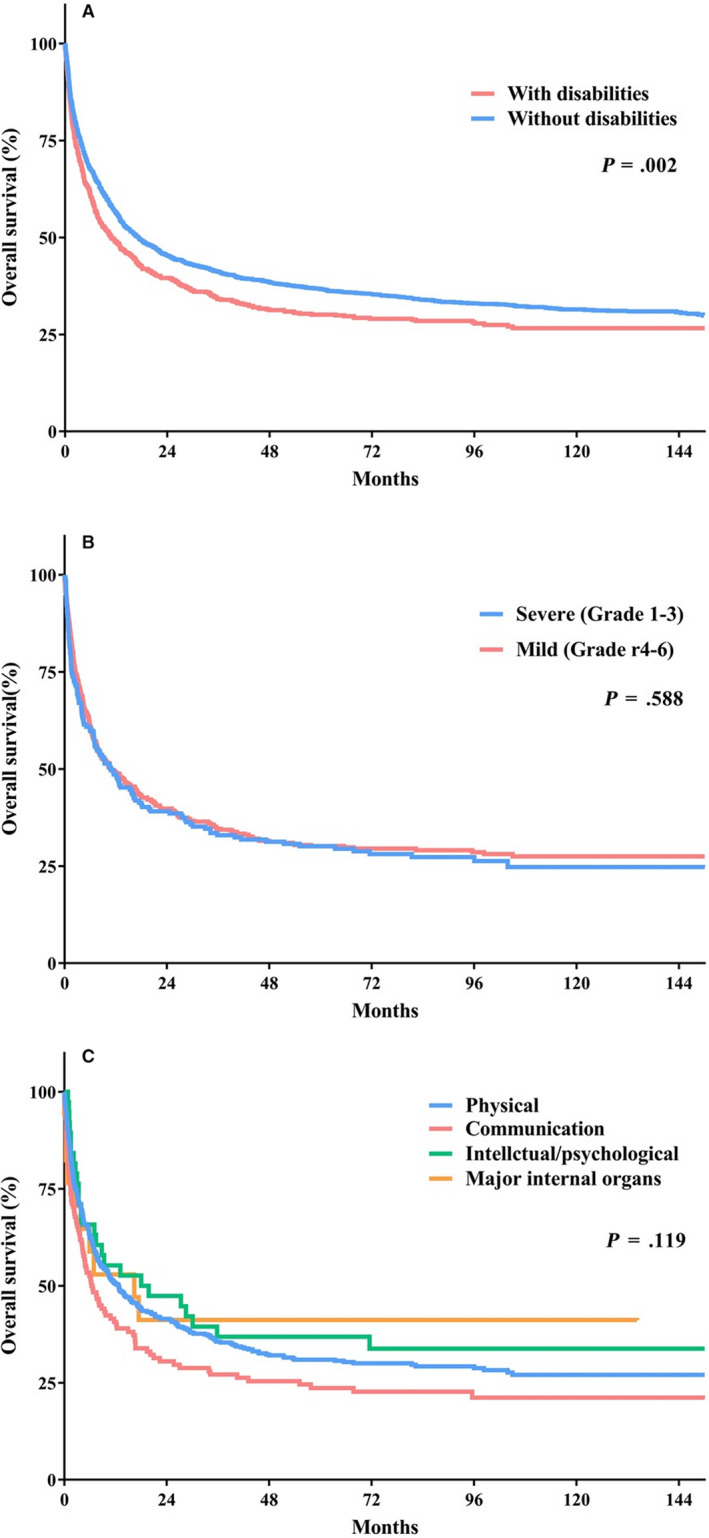
Overall survival curves. A: Comparison by presence of a disability. B: Comparison by severity of disability. C: comparison by type of disability

The survival analysis results are shown in Table 4. The median overall survival (OS) of all patients with AML was 15.7 months (95% confidence interval [CI] 13.794‐17.680). Patients with AML and a disability had a higher mortality risk (median OS 10.8 months, 95% CI 7.567‐14.117) than those without disabilities (median OS 17.1 months, 95% CI 14.672‐19.562, *P* = .002). Disability severity was not associated with overall mortality, but the median OS was nonsignificantly shorter in those with a communication disability (Figure 2).

**Table 4:** Overall survival analysis of AML patients

	Death, N (%)	Median OS (95% CI), mo	Crude HR	Adjust HR	*P*
Patients with AML in whole cohorts	1908 (68.3)	15.7 (0.991‐13.794)	—	—	
Presence of disability					
Patient without disabilities	1501 (67.4)	17.1 (14.672‐19.562)	Reference	Reference	.002
Patients with a disability	407 (71.9)	10.8 (7.567‐14.117)	1.188 (1.064‐1.325)	1.136 (1.016‐1.269)	
Severity of disability					
Severe (grade 1‐3)	131 (73.2)	10.8 (5.401‐16.283)	1.059 (0.860‐1.304)	1.232 (0.996‐1.524)	.588
Mild (grade 4‐6)	276 (71.3)	10.8 (6.538‐15.211)	Reference	Reference	
Type of disability					
Physical	280 (71.2)	12.7 (8.667‐16.762)	Reference	Reference	.119
Communication	92 (78.0)	6.4 (3.459‐9.288)	1.281 (1.012‐1.621)	1.111 (0.869‐1.421)	
Intellectual or psychological	25 (65.8)	18.0 (0.000‐45.195)	0.861 (0.572‐1.297)	1.513 (0.961‐2.383)	
Internal organs	10 (58.9)	16.4 (0.889‐31.899)	0.830 (0.442‐1.559)	0.824 (0.437‐1.553)	

Abbreviations: AML, acute myeloid leukemia; HR, hazard ratio; N, number; OS, overall survival.

## 4 DISCUSSION

This study showed that the frequency of an AML diagnosis is lower in individuals with than in those without a disability. This tendency was more prominent in cases of severe disabilities, major internal organ, and intellectual/psychological disabilities, and in those at low economic levels. The percentage of patients with disabilities who were administered an antileukemic treatment was lower than the percentage of nondisabled patients who received this treatment. Among younger patients (<65 years), fewer patients with disabilities underwent HSCT than did nondisabled patients. The survival rate of patients with AML and disabilities was also inferior compared to that with AML and without a disability.

The diagnostic rate of AML in the present patients with disabilities was significantly lower than that in patients without a disability, and the greater the disability severity was, the greater this tendency also was. There are few previous studies of cancer incidence in individuals with disabilities. In a study of individuals with intellectual disabilities, the age‐standardized incidence of all cancers was not significantly different from that in such patients those without disabilities.^12^ According to Patja et al, there was no significant difference in overall cancer incidence between individuals with disabilities and without disabilities. Only smoking‐related cancers were less common in individuals with disabilities because the smoking rate of individuals with disabilities is lower than that of these patients without disability.^13^ AML is less likely to be caused by external factors except in special cases. Moreover, because the present groups were categorized by sex and age here, differences in the incidence of AML between individuals with and without disabilities cannot be explained demographically. Therefore, our findings suggest that the disability itself may act as a barrier to an AML diagnosis.

For individual patients with disabilities, it could not be determined why their AML diagnosis was delayed. However, some hypotheses can be constructed based on the characteristics of individuals with disabilities. It is possible that people with disabilities did not adequately describe and/or explain the symptoms of AML due to their underlying conditions. This possibility may be especially significant for patients with intellectual/psychological and communication disabilities. People with disabilities also may have poor access to medical facilities that can provide professional care for AML. This is related to physical movement limitations, a lack of awareness of a hematologic malignancy, and a lack of a coping capacity. Finally, caregivers’ decisions may interfere with the rapid management of AML. The lives of individuals with disabilities are often influenced by the caregiver, especially for those with intellectual disabilities, where the disability is severe or rational thinking is difficult. If the caregiver is not aware of the possibility of a hematologic disease in individuals with disabilities, or is passive in management, an AML diagnosis may not be made in timely manner.

It is noteworthy that the AML diagnosis rate of low‐income, nondisabled individuals is higher than the overall average, while the diagnosis rate of low‐income, disabled individuals is the lowest. In Korea, the Korea Health Insurance Corporation is responsible for all necessary medical expenses for those in the lowest income bracket; thus, the burden of the necessary costs associated with an AML diagnosis and subsequent treatment is greatly reduced. This helps reduce the risk that individuals with low incomes will not receive appropriate medical management for fatal diseases. Despite economic support, however, low incomes for people with disabilities were still a major barrier to an AML diagnosis. The current findings suggest that a policy of ensuring only the cost of medical care for people with disabilities is not sufficient to prevent missed AML diagnoses.

Individuals with disabilities are also passive in their treatment of AML. Both the rate at which individuals with disabilities receive chemotherapy for AML and the number of anticancer drugs used were lower than in nondisabled individuals. In younger patients (<65 years), who could normally consider HSCT, the rate of performing HSCT was also low in patients with disabilities. Systemic chemotherapy and HSCT in AML management severely compromise the immune status of recipients and can lead to treatment‐related morbidities. Therefore, it is likely to have been difficult to undertake intensive treatments of individuals with disabilities in a poor overall condition. The treatment rate in the present study was lowest in those with major internal organ and communication disabilities, similar to a study of lung cancer treatments for individuals with disabilities.^7^ Patients with major internal organ disabilities have problems with the vital organ functions required for a cancer treatment; thus, it is understandable that the proportion receiving treatment is relatively low. However, although communication disabilities are not directly related to vital functions, the proportion of such patients receiving treatment was the second lowest after those with major internal organ disabilities. This may stem from the fact that many communication problems in adult patients result from complications of a medical disease. For example, speech impediments are complications of stroke, and visual problems occur as complications of advanced diabetes mellitus. This hypothesis is supported by the fact that the average age of AML patients with communication disabilities is higher than the average age of those with other types of disabilities. In addition, because patients with communication problems may not be able to understand and provide informed consent readily for high‐dose chemotherapy and HSCT, treatment may be more difficult.

In addition to health status, nonmedical problems also affect the prognosis of AML patients with disabilities; economic problems of individuals with disabilities are also a suspected deterrent to providing adequate treatment for AML. Moreover, it is well known that a patient's economic situation is closely related to their prognosis of a malignant disease.^14^, ^15^ Individuals with disabilities are often not employed or are employed part‐time, making it difficult for them to access expensive treatments.^16^ Furthermore, malignant diseases including AML negatively impact the employment or education level of a diagnosed patient.^17^ In individuals with disabilities, who account for a relatively high percentage of lower income individuals, this likely prevents high‐cost treatments such as HSCT from being pursued—more passive treatments can lead to an inferior prognosis. Moreover, whether the disability per se affects the decisions of caregivers and medical teams should be considered. Earlier work reported that the decision to treat leukemia in children was influenced by the socioeconomic environment of their parents and the will of the doctors.^18^ Similarly, it can be assumed that people with disabilities who cannot fully determine their treatment plans will be influenced by their caregivers. In cases where clinicians hesitate to utilize an active treatment or where they exaggerate the risks associated with it, patients and caregivers are affected.

The survival rate of AML patients with disabilities was lower than that of individuals without a disability. In addition to the underlying health problems of patients with disabilities, delayed diagnosis and passive treatment for AML may partially contribute to inferior outcomes. This is similar research results from lung cancer cases in patients with disabilities.^7^ A previous report also suggests that a disability contributes to a lower survival rate of patients with a hematologic malignancy.^19^ When analyzed according to disability type, survival prognosis tended to be poor in those with a communication disability, although the small number of patients did not confirm statistical significance. This occurs because, as mentioned earlier, patients with this type of disability are relatively old and the underlying disease is severe. Unexpectedly, the severity of the disorder was not significantly associated with survival, which is thought to be due to the relatively high proportion of younger patients among the severely disabled here.

Currently, there are various systems for the promotion of the rights and welfare of disabled people. These schemes include life support, rehabilitation, employment support, and vocational training for each type of disability. However, when people with disabilities have severe illnesses, they are more vulnerable than nondisabled people, but additional support policies are inadequate. For fatal diseases such as AML, it is absolutely crucial to recognize the onset immediately and to undertake an active treatment. Therefore, when people with disabilities are diagnosed with a serious disease such as AML, a system should be in place to provide basic economic support and to ensure the mobility of the patient, hospital visits, and long‐term nursing care. For example, a system should be in place to nurture health‐care professionals specialized in treating each type of disability and to allow them to be dedicated to persons with disabilities who are in need of medical treatment. In addition to disabled people, caregivers should also have the means to provide adequate education, economic, and emotional support.

Our research has several limitations. First, this was a retrospective study using big data; it is not possible to obtain detailed personal information such as causes of disabilities, the characteristics of AML, or causes of death. Originally, the prognosis of AML depends on the characteristics of AML itself, such as the presence of a hematologic disease before the AML diagnosis, the subtype of AML, the presence/absence of chromosome abnormalities, and the mutation profile. Depending on these characteristics, the intensity of treatment may differ. Our study could not determine whether there was a difference in the disease characteristics of AML with or without a disability present due to methodological limitations. Thus, the poor prognosis of AML in people with disabilities is not entirely excluded from the possibility that it is due to the nature of the disease itself, not to external factors such as the timing of diagnosis or the intensity of treatment. Another limitation of our study was that we were unable to determine the type or intensity of the combination therapy because the treatment information was extracted from claims information for insurance benefits. As a result, the use of low‐intensity anticancer drugs for conventional purposes was considered here as a case of chemotherapy, and the proportion of people receiving chemotherapy may be higher than previously known. However, patients who are aged or in a poor general condition often do not use anticancer drugs at all; therefore, even if it is a temporary low‐intensity regimen, determining whether or not they have been prescribed may be appropriate for evaluating the aggressiveness of a treatment. In addition, this analysis did not include information about anticancer drugs or treatments not covered by medical insurance. Accordingly, care is needed when interpreting the results, as drugs provided through clinical trials and the effects of treatments paid by the patient are not included. Finally, due to data access limitations, we could not obtain information about the number of disabilities that each patient had. It is possible that more patients with multiple disabilities have more disadvantages with regard to an AML prognosis. In this study, only one major disability for each patient was analyzed. In order to establish AML treatment strategies that take into account the characteristics of disabled patients more accurately, further research based on more detailed patient classification techniques is necessary.

Nonetheless, to the best of our knowledge, this is the first study to use data from a large national registry to analyze the AML diagnosis and treatment patterns of individuals with disabilities. A disability is an obstacle preventing proper treatment of AML for a variety of reasons; thus, efforts to overcome the adverse effects of a disability are necessary. In addition, it is essential to consider measures for economic problems and education, as well as social support for patients and caregivers. Further research is needed to develop public health policies for patients with AML and disabilities.

## CONFLICT OF INTEREST

None declared.

## AUTHOR CONTRIBUTIONS

Study design: Jihyun Kwon, So Young Kim and Jong Hyock Park; Investigation: Kyoung Eun Yeob, So Young Kim and Jong Hyock Park; Resources: Yeon‐Yong Kim, Jong Heon Park and Jong Hyock Park; Analysis and interpretation: Kyoung Eun Yeob; Writing of manuscript: Jihyun Kwon and So Young Kim; Review & Editing: Hye Sook Han, Ki Hyeong Lee, Dong Wook Shin, Jong Hyock Park and Ichiro Kawachi; Supervision: Jihyun Kwon, So Young Kim, Jong Hyock Park and Ichiro Kawachi.

## ETHICAL APPROVAL

This article does not contain any studies with human participants or animals performed by any of the authors.

## Supporting information

Supplementary Material

## Data Availability

Data sharing is not applicable to this article due to technical or time limitations.
