# Supplementary material for: Differences in diagnosis, treatment, and survival rate of acute myeloid leukemia with or without disabilities: A national cohort study in the Republic of Korea
Source: Cancer Med. 2020 Jun 3;9(15):5335–44. doi: 10.1002/cam4.3179 (PMC7402831; doi:10.1002/cam4.3179)
Supplement: Supplementary file 1 — Supplementary Material [file CAM4-9-5335-s001.docx]

| Category | Type of disability |
| --- | --- |
| Physical | amputation, motor disturbance, joint disability, disfigurements or deformities, spinal cord injury, brain impairment |
| Communication | sight, hearing or speech problems |
| Intellectual or psychological | difficulty learning or understanding things, mental illness |
| Major internal organ | functional impairment of internal organ diseases such as kidney, heart, liver, lung, intestine |
| **Supplementary Table 1. Categorization of type of disabilities** | |

|  |  | All cohort | People without disabilities | People with disabilities |
| --- | --- | --- | --- | --- |
|  |  | N (%) | N (%) | N (%) |
|  |  | 11,115,800(100.0) | 8,329,350 (100.0) | 2,776,450 (100.0) |
| **Age** | <65 | 7,170,394 (64.5) | 5,322,371 (63.9) | 1,848,023 (66.6) |
|  | ≥65 | 3,640,063 (32.7) | 2,719,784 (32.7) | 920,279 (33.1) |
|  | No data | 305,343 (2.7) | 287,195 (3.4) | 88,148 (0.3) |
| **Sex** | Male | 6,498,991 (58.5) | 4,874,242 (58.5) | 1,624,749 (58.5) |
|  | Female | 4,606,809 (41.4) | 3,455,108 (41.5) | 1,151,701 (41.5) |
|  | No data | 10,000 (0.1) | 0 (0.0) | 0 (0.0) |
| **Economic status** | Below poverty line (lowest) | 791,356 (7.1) | 323,466 (3.9) | 467,890 (16.9) |
|  | 1^st^ quartile | 1,985,606 (17.9) | 1,471,840 (17.7) | 513,766 (18.5) |
|  | 2^nd^ quartile | 1,923,186 (17.3) | 1,459,623 (17.5) | 463,563 (16.7) |
|  | 3^rd^ quartile | 2,505,234 (22.5) | 1,929,692 (23.2) | 575,542 (20.7) |
|  | 4^th^ quartile (highest) | 3,605,075 (32.4) | 2,857,534 (34.3) | 747,541 (26.9) |
|  | No data | 305,343 (2.7) | 287,195 (3.4) | 8,148 (0.3) |
| **Severity of disability** | Severe (grade 1-3) | - | - | 1,091,794 (39.3) |
|  | Mild (grade 4-6) | - | - | 1,684,656 (60.7) |
|  | No data |  |  | 0 (0.0) |
| **Type of disability** | Physical | - | - | 1,728,916 (62.3) |
|  | Communication | - | - | 588,712 (21.2) |
|  | Intellectual or psychological | - | - | 307,720 (11.1) |
|  | Major internal organs | - | - | 151,102 (5.44) |
|  | No data |  |  | 0 (0.0) |
| **Supplementay Table 2. Basic characteristics of people in entire cohort.** | | | | |

|  |  | Severity of disability | | Type of disability | | | |
| --- | --- | --- | --- | --- | --- | --- | --- |
|  |  | Severe  (n=1,091,794) | Mild  (n=1,684,656) | Physical  (n=1,728,916) | Communication  (n=588,712) | Intellectual or psychological  (n=307,720) | Major internal organs  (n=151,102) |
|  |  | N (%) | N (%) | N (%) | N (%) | N (%) | N (%) |
| **Age** | <65 | 785,173 (71.9) | 1,071,018 (63.6) | 1,126,472 (65.2) | 322,506 (54.8) | 295,482 (96.0) | 106,278 (70.3) |
|  | ≥65 | 306,621 (28.1) | 613,638 (36.4) | 597,370 (34.6) | 264,479 (44.9) | 11,332 (3.7) | 44,383 (29.4) |
| **Sex** | Male | 653,958 (59.9) | 970,790 (57.6) | 1,006,246 (58.2) | 341,035 (57.9) | 182,473 (59.3) | 95,216 (63.0) |
|  | Female | 437,836 (40.1) | 713,866 (42.4) | 722,699 (41.8) | 244,936 (41.6) | 125,239 (40.7) | 58,606 (38.8) |
| **Income** | Below poverty line (lowest) | 306,704 (28.1) | 161,187 (9.6) | 223,034 (12.9) | 75,590 (12.8) | 140,933 (45.8) | 27,995 (18.5) |
|  | I | 177,224 (16.2) | 344,708 (20.5) | 337,144 (19.5) | 115,436 (19.6) | 43,387 (14.1) | 26,765 (17.7) |
|  | II | 157,095 (14.4) | 306,468 (18.2) | 302,565 (17.5) | 101,373 (17.2) | 35,079 (11.4) | 24,612 (16.3) |
|  | III | 197,495 (18.1) | 378,033 (22.4) | 378,640 (21.9) | 124,226 (21.1) | 40,618 (13.2) | 31,534 (20.9) |
|  | IV (highest) | 253,275 (23.2) | 494,259 (29.3) | 487,562 (28.2) | 169,346 (28.8) | 47,695 (15.5) | 42,916 (28.4) |
| **Supplementary Table 3. Basic characteristics of people in cohort of disability in study cohort.** | | | | | | | |
